# Supplementary material for: Identifying transcriptomic correlates of histology using deep learning
Source: PLoS One. 2020 Nov 25;15(11):e0242858. doi: 10.1371/journal.pone.0242858 (PMC7688140; doi:10.1371/journal.pone.0242858)

S1 Fig. Histograms of features, genes and their correlation. (A) Histogram of feature values. (B) Histogram of log_2 transformed gene expression values $log_2(1+g)$. (C) Histogram of gene-feature correlations (for genes with highest median tissue log_2 expression over 10). (D) Histogram of gene-feature correlations separately for non-negative features (e.g. outputs of ReLU units) and potentially negative features.

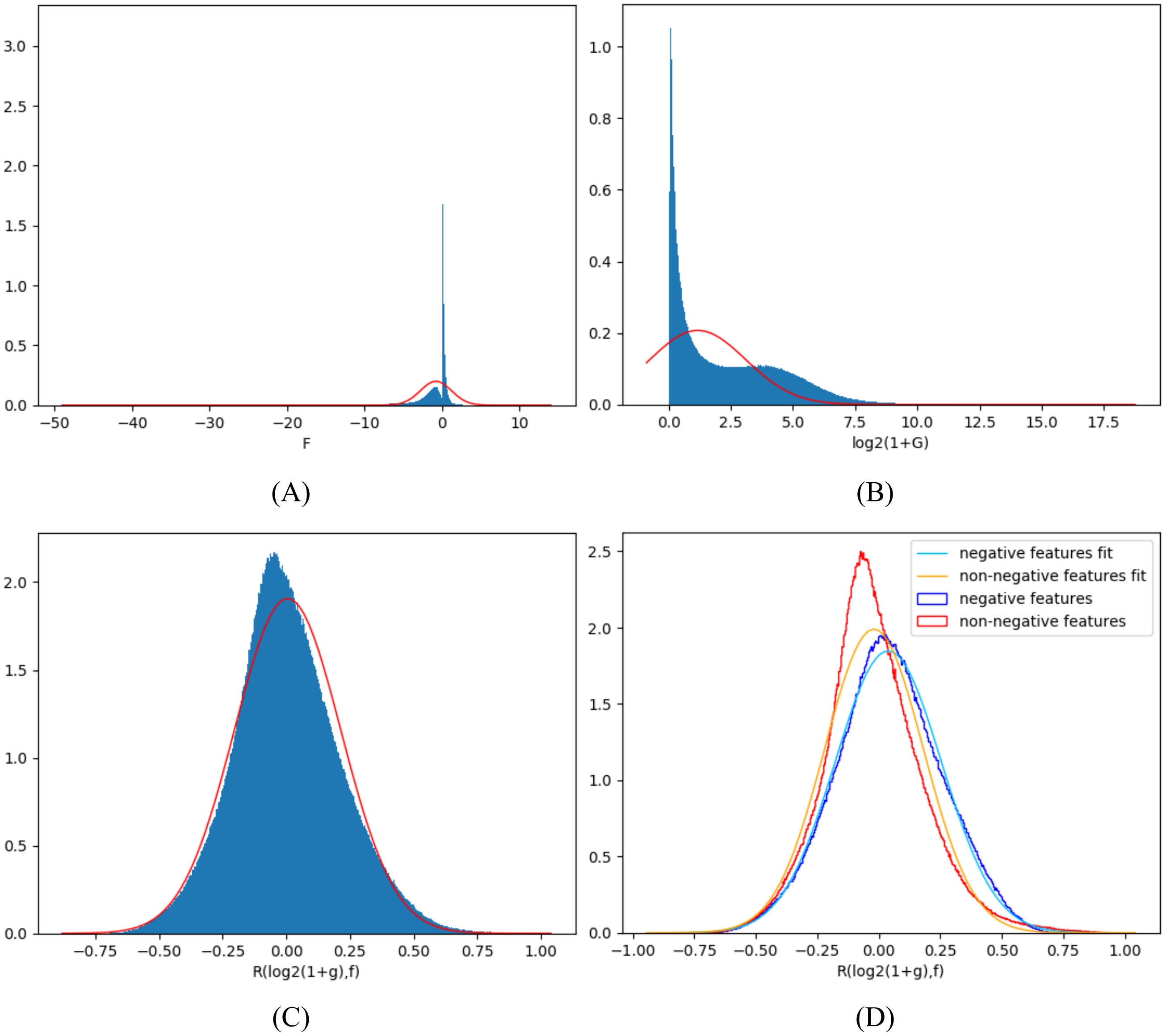

Supplement: S1 Fig — (A) Histogram of feature values. (B) Histogram of log2 transformed gene expression values log2(1+g). (C) Histogram of gene-feature correlations (for genes with highest median tissue log2 expression over 10). (D) Histogram of gene-feature correlations separately for non-negative features (e.g. outputs of ReLU units) and potentially negative features. (PDF) [file pone.0242858.s001.pdf]
