# Supplementary material for: Identifying transcriptomic correlates of histology using deep learning
Source: PLoS One. 2020 Nov 25;15(11):e0242858. doi: 10.1371/journal.pone.0242858 (PMC7688140; doi:10.1371/journal.pone.0242858)
Supplement: S2 Fig — (A) Numbers of correlated genes for individual features. Features are sorted in decreasing order of the corresponding numbers of correlated genes. (B) Numbers of correlated features per gene. Genes are sorted in decreasing order of the corresponding numbers of correlated features. Various correlation thresholds are applied (from 0.7 to 0.95). (PDF) [file pone.0242858.s002.pdf]

S2 Fig. Numbers of correlated genes for individual features and respectively correlated features per gene. (A) Numbers of correlated genes for individual features. Features are sorted in decreasing order of the corresponding numbers of correlated genes. (B) Numbers of correlated features per gene. Genes are sorted in decreasing order of the corresponding numbers of correlated features. Various correlation thresholds are applied (from 0.7 to 0.95).

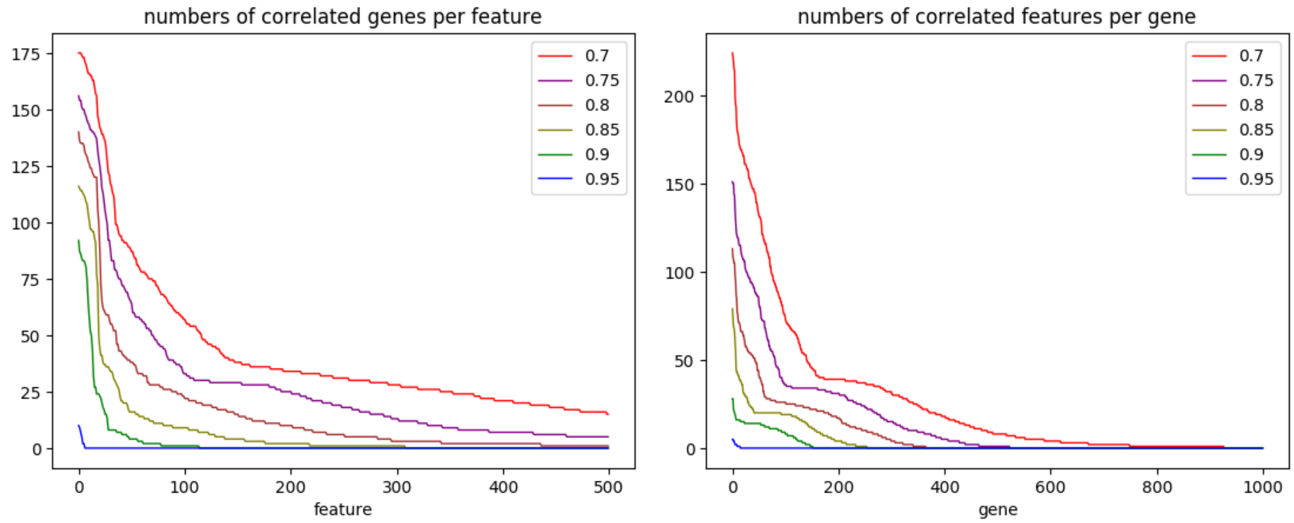

(A)

(B)
