# Supplementary material for: Identifying transcriptomic correlates of histology using deep learning
Source: PLoS One. 2020 Nov 25;15(11):e0242858. doi: 10.1371/journal.pone.0242858 (PMC7688140; doi:10.1371/journal.pone.0242858)
Supplement: S3 Fig — Scatter plots of gene-feature correlations computed on the validation- and respectively test dataset. (A) scatter plot of correlations (R = 0.9205). (B) scatter plot of Fisher transformed correlations (R = 0.9233). (PDF) [file pone.0242858.s003.pdf]

S3 Fig. Reproducibility of gene-feature correlations between datasets. Scatter plots of gene-feature correlations computed on the validation- and respectively test dataset. (A) scatter plot of *correlations* (R=0.9205). (B) scatter plot of *Fisher transformed* correlations (R=0.9233)

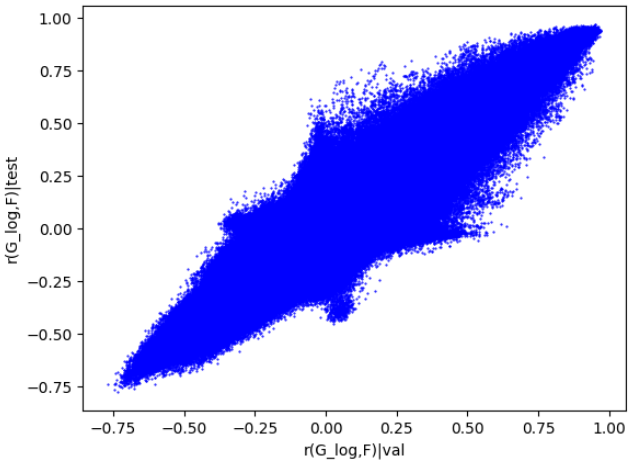

(A)

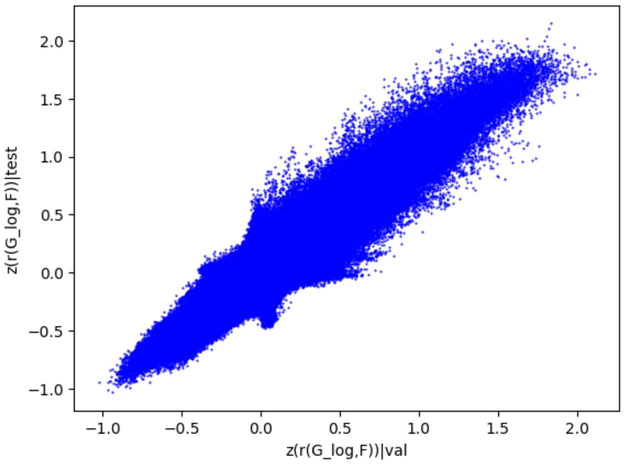

(B)
