# Supplementary material for: Identifying transcriptomic correlates of histology using deep learning
Source: PLoS One. 2020 Nov 25;15(11):e0242858. doi: 10.1371/journal.pone.0242858 (PMC7688140; doi:10.1371/journal.pone.0242858)
Supplement: S1 Table — (PDF) [file pone.0242858.s004.pdf]

S1 Table. The numbers of slides and respectively tiles for each data set and tissue type.

Tissue	slides				tiles			
	train	*validation*	*test*	*total*	*train*	*validation*	*test*	*total*
Adipose - Subcutaneous	22	7	7	36	6,915	2,203	2,318	11,436
Adipose - Visceral (Omentum)	12	5	4	21	3,374	1,668	1,008	6,050
Adrenal Gland	29	9	9	47	10,007	3,382	2,040	15,429
Artery - Aorta	25	8	9	42	7,346	2,203	2,646	12,195
Artery - Coronary	30	10	10	50	1,559	816	703	3,078
Artery - Tibial	27	9	9	45	1,581	687	529	2,797
Bladder	7	2	2	11	4,472	1,066	1,276	6,814
Brain - Cerebellum	29	10	10	49	7,449	2,848	1,958	12,255
Brain - Cortex	30	9	10	49	8,793	2,495	2,845	14,133
Breast - Mammary Tissue	25	9	8	42	7,417	3,739	3,625	14,781
Cervix - Ectocervix	3	2	1	6	1,161	908	823	2,892
Cervix - Endocervix	3	1	1	5	1,375	221	597	2,193
Colon - Sigmoid	30	10	10	50	12,413	3,078	4,067	19,558
Colon - Transverse	28	9	10	47	9,754	3,698	3,159	16,611
Esophagus - Gastroesophageal Junction	28	10	10	48	13,195	5,032	5,800	24,027
Esophagus - Mucosa	28	10	10	48	6,225	2,086	1,937	10,248
Esophagus - Muscularis	28	8	9	45	12,076	3,246	3,612	18,934
Fallopian Tube	4	1	2	7	638	214	170	1,022
Heart - Atrial Appendage	30	10	10	50	10,691	3,833	4,337	18,861
Heart - Left Ventricle	29	9	9	47	10,861	3,021	2,772	16,654
Kidney - Cortex	27	9	9	45	10,139	3,227	3,365	16,731
Liver	30	10	10	50	13,229	4,111	4,197	21,537
Lung	30	10	9	49	10,314	3,181	3,278	16,773
Minor Salivary Gland	30	10	10	50	4,426	1,527	1,878	7,831
Muscle - Skeletal	30	10	10	50	12,499	3,732	4,059	20,290
Nerve - Tibial	29	9	9	47	3,359	1,365	789	5,513
Ovary	29	10	9	48	12,178	3,475	3,594	19,247
Uterus	29	10	10	49	12,229	4,281	4,025	20,535
Vagina	30	10	10	50	16,274	5,171	5,312	26,757
Pancreas	29	9	10	48	12,863	3,965	4,799	21,627
Pituitary	30	10	9	49	4,940	1,598	1,793	8,331
Prostate	29	9	10	48	13,939	3,718	4,792	22,449
Skin - Not Sun Exposed	30	10	10	50	13,990	4,644	4,128	22,762
Skin - Sun Exposed	30	10	10	50	11,582	4,911	3,545	20,038
Small Intestine - Terminal Ileum	29	10	10	49	10,455	3,158	3,375	16,988
Spleen	30	9	10	49	12,747	4,595	4,530	21,872
Stomach	30	10	9	49	16,753	4,689	3,799	25,241
Testis	30	8	10	48	9,556	2,891	3,627	16,074
Thyroid	28	9	10	47	10,566	3,440	4,918	18,924
Total	1,006	330	334	1,670	349,340	114,123	116,025	579,488
