# Supplementary material for: Identifying transcriptomic correlates of histology using deep learning
Source: PLoS One. 2020 Nov 25;15(11):e0242858. doi: 10.1371/journal.pone.0242858 (PMC7688140; doi:10.1371/journal.pone.0242858)
Supplement: S2 Table — Genes are grouped w.r.t. tissues and ordered by correlation—for each gene we only show the best correlated feature, in the form [layer]_[channel] (for the architecture VGG16 and the test dataset; correlation threshold = 0.75, log2 gene expression threshold = 7). (PDF) [file pone.0242858.s005.pdf]

S2 Table. Genes correlated with visual histopathological features. Genes are grouped w.r.t. tissues and ordered by correlation - for each gene we only show the best correlated feature, in the form [layer]_[channel] (for the architecture VGG16 and the test dataset; correlation threshold=0.75, *log₂* gene expression threshold=7).

Gene	Gene name	Tissue with highest expression	Highest median tissue expression (log_2)	Best correlated feature	r
FABP4	fatty acid binding protein 4	Adipose - Visceral (Omentum)	12.6	1_14	0.767
CYP11B1	cytochrome P450 family 11 subfamily B member 1	Adrenal Gland	11.7	20_467	0.852
CYP17A1	cytochrome P450 family 17 subfamily A member 1	Adrenal Gland	12.4	20_467	0.805
HSD3B2	hydroxy-delta-5-steroid dehydrogenase, 3 beta- and steroid delta-isomerase 2	Adrenal Gland	11.2	29_123	0.791
CYP21A2	cytochrome P450 family 21 subfamily A member 2	Adrenal Gland	10.7	29_123	0.770
STAR	steroidogenic acute regulatory protein	Adrenal Gland	12.5	29_132	0.770
CYP11A1	cytochrome P450 family 11 subfamily A member 1	Adrenal Gland	9.5	29_148	0.755
TPM4	tropomyosin 4	Artery - Aorta	9.1	19_468	0.767
S100A6	S100 calcium binding protein A6	Artery - Aorta	11.6	19_162	0.760
YAP1	Yes1 associated transcriptional regulator	Artery - Aorta	7.6	19_162	0.758
MARVELD1	MARVEL domain containing 1	Artery - Tibial	7.8	19_162	0.764
PTRF	caveolae associated protein 1	Artery - Tibial	10.1	14_129	0.763
GNG12	G protein subunit gamma 12	Artery - Tibial	7.1	12_209	0.755
GABRA6	gamma-aminobutyric acid type A receptor subunit alpha6	Brain - Cerebellum	7.5	29_447	0.949
ZIC4	Zic family member 4	Brain - Cerebellum	7.0	29_447	0.922
RP11-547D24.1		Brain - Cerebellum	7.2	29_479	0.916
NEUROD2	neuronal differentiation 2	Brain - Cerebellum	7.2	29_463	0.915
GRM4	glutamate metabotropic receptor 4	Brain - Cerebellum	8.3	29_447	0.913
LINC00599	MIR124-1 host gene	Brain - Cerebellum	10.0	29_463	0.907
NEUROD1	neuronal differentiation 1	Brain - Cerebellum	7.8	29_479	0.873
CRTAM	cytotoxic and regulatory T cell molecule	Brain - Cerebellum	7.2	29_479	0.868
KCNJ9	potassium inwardly rectifying channel subfamily J member 9	Brain - Cerebellum	7.1	29_463	0.858
TTC9B	tetratricopeptide repeat domain 9B	Brain - Cerebellum	7.2	27_140	0.856
ZIC2	Zic family member 2	Brain - Cerebellum	7.9	29_463	0.854
GNG13	G protein subunit gamma 13	Brain - Cerebellum	7.2	29_463	0.854
SLC12A5	solute carrier family 12 member 5	Brain - Cerebellum	7.5	27_140	0.850
ZIC1	Zic family member 1	Brain - Cerebellum	8.1	29_479	0.844
TMEM151B	transmembrane protein 151B	Brain - Cerebellum	7.7	27_140	0.844
ELAVL3	ELAV like RNA binding protein 3	Brain - Cerebellum	8.0	29_463	0.834
DIRAS2	DIRAS family GTPase 2	Brain - Cerebellum	7.6	27_140	0.831
C16orf11	proline rich 35	Brain - Cerebellum	7.6	29_447	0.810
GABRD	gamma-aminobutyric acid type A receptor subunit delta	Brain - Cerebellum	9.1	29_463	0.807
GFAP	glial fibrillary acidic protein	Brain - Cerebellum	9.7	29_485	0.804
CACNA1A	calcium voltage-gated channel subunit alpha1 A	Brain - Cerebellum	8.2	27_140	0.794
PVALB	parvalbumin	Brain - Cerebellum	9.1	29_463	0.781
HPCAL4	hippocalcin like 4	Brain - Cerebellum	7.5	29_463	0.781
RP11-981G7.6		Brain - Cerebellum	7.1	30_479	0.770
TMEM145	transmembrane protein 145	Brain - Cerebellum	8.2	25_302	0.766
MAP6D1	MAP6 domain containing 1	Brain - Cerebellum	7.1	29_463	0.765
CPLX2	complexin 2	Brain - Cerebellum	8.7	27_140	0.762
SPOCK2	SPARC (osteonectin), cwcv and kazal like domains proteoglycan 2	Brain - Cerebellum	8.6	27_148	0.761
AGAP2	ArfGAP with GTPase domain, ankyrin repeat and PH domain 2	Brain - Cerebellum	8.0	29_463	0.761
MLC1	modulator of VRAC current 1	Brain - Cerebellum	7.3	29_485	0.760
ATP6V1G2	ATPase H+ transporting V1 subunit G2	Brain - Cerebellum	9.2	29_463	0.752
TUBB4A	tubulin beta 4A class IVa	Brain - Cerebellum	9.5	27_488	0.752
SLC1A2	solute carrier family 1 member 2	Brain - Cortex	8.7	18_8	0.844
SNCB	synuclein beta	Brain - Cortex	9.2	29_485	0.814
GRIN1	glutamate ionotropic receptor NMDA type subunit 1	Brain - Cortex	7.9	18_8	0.812
HPCA	hippocalcin	Brain - Cortex	7.5	29_485	0.811

Gene	Gene name	Tissue with highest expression	Highest median tissue expression (log_2)	Best correlated feature	r
PACSIN1	protein kinase C and casein kinase substrate in neurons 1	Brain - Cortex	8.5	29_463	0.810
SLC17A7	solute carrier family 17 member 7	Brain - Cortex	9.3	18_8	0.799
CAMK2A	calcium/calmodulin dependent protein kinase II alpha	Brain - Cortex	9.2	23_417	0.786
CABP1	calcium binding protein 1	Brain - Cortex	7.3	30_231	0.779
DDN	dendrin	Brain - Cortex	7.9	25_478	0.775
RAPGEF4	Rap guanine nucleotide exchange factor 4	Brain - Cortex	7.6	18_224	0.767
KCNH3	potassium voltage-gated channel subfamily H member 3	Brain - Cortex	7.2	20_380	0.767
CAMKV	CaM kinase like vesicle associated	Brain - Cortex	7.6	18_8	0.766
NSG2	neuronal vesicle trafficking associated 2	Brain - Cortex	7.3	27_278	0.758
CEND1	cell cycle exit and neuronal differentiation 1	Brain - Cortex	8.6	29_485	0.755
KIF5A	kinesin family member 5A	Brain - Cortex	10.0	29_485	0.754
GNG3	G protein subunit gamma 3	Brain - Cortex	8.1	27_278	0.753
SUMO2	small ubiquitin like modifier 2	Cervix - Ectocervix	7.8	19_468	0.771
DDAH2	dimethylarginine dimethylaminohydrolase 2	Cervix - Ectocervix	7.8	19_162	0.765
CYBRD1	cytochrome b reductase 1	Cervix - Ectocervix	8.7	19_162	0.763
NBL1	NBL1, DAN family BMP antagonist	Cervix - Ectocervix	9.6	19_162	0.758
CRTAP	cartilage associated protein	Cervix - Ectocervix	8.1	12_223	0.756
HNRNPUL1	heterogeneous nuclear ribonucleoprotein U like 1	Cervix - Ectocervix	7.2	19_468	0.754
HMGB1	high mobility group box 1	Cervix - Ectocervix	7.4	19_468	0.753
PTMA	prothymosin alpha	Cervix - Endocervix	10.1	19_468	0.802
PIAS3	protein inhibitor of activated STAT 3	Cervix - Endocervix	7.0	19_468	0.764
SPIN1	spindlin 1	Cervix - Endocervix	7.2	19_333	0.752
PLXNB2	plexin B2	Cervix - Endocervix	8.0	12_164	0.750
TACR2	tachykinin receptor 2	Colon - Sigmoid	7.8	23_97	0.753
MANBAL	mannosidase beta like	Fallopian Tube	7.0	21_493	0.752
NKX2-5	NK2 homeobox 5	Heart - Atrial Appendage	7.0	30_214	0.903
BMP10	bone morphogenetic protein 10	Heart - Atrial Appendage	8.3	29_313	0.855
NPPA	natriuretic peptide A	Heart - Atrial Appendage	14.9	29_481	0.777
MYL4	myosin light chain 4	Heart - Atrial Appendage	11.2	29_6	0.755
TECRL	trans-2,3-enoyl-CoA reductase like	Heart - Atrial Appendage	7.1	11_75	0.753
NMRK2	nicotinamide riboside kinase 2	Heart - Atrial Appendage	9.8	30_72	0.751
MYBPC3	myosin binding protein C3	Heart - Left Ventricle	10.8	23_270	0.783
TNNI3	troponin I3, cardiac type	Heart - Left Ventricle	12.0	25_28	0.780
XIRP1	xin actin binding repeat containing 1	Heart - Left Ventricle	7.4	30_72	0.770
TNNT2	troponin T2, cardiac type	Heart - Left Ventricle	11.6	25_31	0.759
CSRP3	cysteine and glycine rich protein 3	Heart - Left Ventricle	9.4	30_72	0.755
AQP2	aquaporin 2	Kidney - Cortex	7.6	29_280	0.890
MIOX	myo-inositol oxygenase	Kidney - Cortex	7.1	29_488	0.888
UMOD	uromodulin	Kidney - Cortex	7.4	27_274	0.874
NAT8	N-acetyltransferase 8 (putative)	Kidney - Cortex	7.2	30_20	0.827
CDH16	cadherin 16	Kidney - Cortex	8.7	30_488	0.816
FXYD4	FXYD domain containing ion transport regulator 4	Kidney - Cortex	7.2	30_280	0.782
F9	coagulation factor IX	Liver	8.1	29_192	0.975
C8A	complement C8 alpha chain	Liver	8.0	29_192	0.973
HAO1	hydroxyacid oxidase 1	Liver	8.0	29_192	0.968
CFHR2	complement factor H related 2	Liver	8.2	29_192	0.966
C9	complement C9	Liver	8.8	29_192	0.962
PLG	plasminogen	Liver	8.7	29_234	0.959
C3P1	complement component 3 precursor pseudogene	Liver	7.8	29_192	0.957
AKR1C4	aldo-keto reductase family 1 member C4	Liver	7.2	29_192	0.956
APOF	apolipoprotein F	Liver	7.2	29_192	0.956
SLC25A47	solute carrier family 25 member 47	Liver	8.6	29_234	0.954
APOA5	apolipoprotein A5	Liver	8.8	29_192	0.954
SERPINC1	serpin family C member 1	Liver	10.1	29_192	0.952
HRG	histidine rich glycoprotein	Liver	9.2	29_234	0.952
F2	coagulation factor II, thrombin	Liver	9.4	29_192	0.949

Gene	Gene name	Tissue with highest expression	Highest median tissue expression (log_2)	Best correlated feature	r
APOC4	apolipoprotein C4	Liver	7.8	29_192	0.949
SERPINA10	serpin family A member 10	Liver	7.6	29_192	0.948
AHSG	alpha 2-HS glycoprotein	Liver	10.7	29_192	0.946
AFM	afamin	Liver	7.9	29_234	0.946
APCS	amyloid P component, serum	Liver	10.7	29_192	0.942
C8B	complement C8 beta chain	Liver	8.2	29_192	0.941
AGXT	alanine--glyoxylate and serine--pyruvate aminotransferase	Liver	10.9	29_234	0.939
CYP8B1	cytochrome P450 family 8 subfamily B member 1	Liver	8.1	29_234	0.938
BAAT	bile acid-CoA:amino acid N-acyltransferase	Liver	7.7	29_192	0.938
ITIH1	inter-alpha-trypsin inhibitor heavy chain 1	Liver	9.1	29_192	0.937
ANGPTL3	angiopoietin like 3	Liver	7.3	29_234	0.934
SLC22A1	solute carrier family 22 member 1	Liver	9.1	30_192	0.933
APOA2	apolipoprotein A2	Liver	12.2	29_192	0.931
CYP4A11	cytochrome P450 family 4 subfamily A member 11	Liver	8.3	29_234	0.924
CPB2	carboxypeptidase B2	Liver	8.5	29_192	0.922
ACSM2A	acyl-CoA synthetase medium chain family member 2A	Liver	7.4	23_292	0.920
PROC	protein C, inactivator of coagulation factors Va and VIIIa	Liver	7.9	29_234	0.919
APOH	apolipoprotein H	Liver	11.7	29_234	0.919
ACSM2B	acyl-CoA synthetase medium chain family member 2B	Liver	7.4	23_292	0.919
SLC22A7	solute carrier family 22 member 7	Liver	7.7	29_234	0.900
CPN2	carboxypeptidase N subunit 2	Liver	7.3	29_234	0.900
KNG1	kininogen 1	Liver	8.6	30_234	0.897
FGB	fibrinogen beta chain	Liver	12.6	29_192	0.896
HPX	hemopexin	Liver	11.1	30_192	0.895
CYP2A6	cytochrome P450 family 2 subfamily A member 6	Liver	9.6	29_192	0.888
ITIH2	inter-alpha-trypsin inhibitor heavy chain 2	Liver	7.7	29_192	0.885
PON1	paraoxonase 1	Liver	7.2	18_279	0.885
TAT	tyrosine aminotransferase	Liver	9.2	29_192	0.884
FGL1	fibrinogen like 1	Liver	10.2	18_231	0.879
SERPINA11	serpin family A member 11	Liver	7.8	29_192	0.877
AMBP	alpha-1-microglobulin/bikunin precursor	Liver	11.7	18_231	0.876
G6PC	glucose-6-phosphatase catalytic subunit	Liver	7.1	29_234	0.872
GC	GC vitamin D binding protein	Liver	9.8	29_192	0.867
PAH	phenylalanine hydroxylase	Liver	8.2	22_418	0.863
SULT2A1	sulfotransferase family 2A member 1	Liver	8.6	29_30	0.863
APOC1P1	apolipoprotein C1 pseudogene 1	Liver	7.0	30_30	0.859
FGA	fibrinogen alpha chain	Liver	12.2	30_192	0.858
ASGR2	asialoglycoprotein receptor 2	Liver	8.8	30_192	0.854
CRP	C-reactive protein	Liver	12.6	29_192	0.852
VTN	vitronectin	Liver	11.2	18_279	0.851
TTR	transthyretin	Liver	11.1	18_231	0.850
SLC2A2	solute carrier family 2 member 2	Liver	7.2	29_234	0.849
SLC13A5	solute carrier family 13 member 5	Liver	8.4	27_275	0.845
BHMT	betaine--homocysteine S-methyltransferase	Liver	8.5	23_292	0.844
APOC3	apolipoprotein C3	Liver	13.0	29_192	0.840
ALB	albumin	Liver	15.1	18_231	0.840
ADH1A	alcohol dehydrogenase 1A (class I), alpha polypeptide	Liver	8.0	29_192	0.835
TDO2	tryptophan 2,3-dioxygenase	Liver	7.7	30_205	0.834
CYP2B6	cytochrome P450 family 2 subfamily B member 6	Liver	7.3	29_234	0.834
SLC27A5	solute carrier family 27 member 5	Liver	8.0	29_192	0.832
C8G	complement C8 gamma chain	Liver	8.7	30_192	0.831
RDH16	retinol dehydrogenase 16	Liver	7.9	30_75	0.830
ORM1	orosomucoid 1	Liver	12.5	30_192	0.829
FGG	fibrinogen gamma chain	Liver	12.2	30_192	0.829
TFR2	transferrin receptor 2	Liver	8.8	27_275	0.828
ORM2	orosomucoid 2	Liver	11.3	30_192	0.826

Gene	Gene name	Tissue with highest expression	Highest median tissue expression (*log2*)	Best correlated feature	r
C4BPA	complement component 4 binding protein alpha	Liver	8.4	27_380	0.823
CYP2C8	cytochrome P450 family 2 subfamily C member 8	Liver	9.5	23_492	0.821
CFHR1	complement factor H related 1	Liver	8.1	29_192	0.821
DPYS	dihydropyrimidinase	Liver	7.1	18_249	0.819
UGT2B7	UDP glucuronosyltransferase family 2 member B7	Liver	7.2	23_292	0.819
CYP2E1	cytochrome P450 family 2 subfamily E member 1	Liver	11.1	30_192	0.817
SERPIND1	serpin family D member 1	Liver	7.6	29_192	0.810
SERPINA6	serpin family A member 6	Liver	7.7	29_234	0.806
MAT1A	methionine adenosyltransferase 1A	Liver	9.7	20_471	0.806
F12	coagulation factor XII	Liver	8.7	30_75	0.804
IGFBP1	insulin like growth factor binding protein 1	Liver	7.2	29_75	0.802
ASGR1	asialoglycoprotein receptor 1	Liver	8.7	27_275	0.798
ADH4	alcohol dehydrogenase 4 (class II), pi polypeptide	Liver	9.1	29_192	0.787
APOB	apolipoprotein B	Liver	8.5	30_438	0.784
SLC38A4	solute carrier family 38 member 4	Liver	7.3	29_192	0.780
SAA4	serum amyloid A4, constitutive	Liver	10.4	29_192	0.778
CREB3L3	cAMP responsive element binding protein 3 like 3	Liver	8.2	20_481	0.777
CPS1	carbamoyl-phosphate synthase 1	Liver	8.6	29_192	0.775
SDS	serine dehydratase	Liver	8.9	27_275	0.769
ARG1	arginase 1	Liver	9.0	20_194	0.768
CYP2C9	cytochrome P450 family 2 subfamily C member 9	Liver	8.6	20_481	0.768
A1BG	alpha-1-B glycoprotein	Liver	8.9	30_192	0.768
HPD	4-hydroxyphenylpyruvate dioxygenase	Liver	10.1	30_234	0.761
APOM	apolipoprotein M	Liver	7.2	29_234	0.753
SFTPA1	surfactant protein A1	Lung	10.4	30_155	0.849
SFTPC	surfactant protein C	Lung	9.8	30_155	0.838
SFTPA2	surfactant protein A2	Lung	10.6	30_155	0.803
SCGB3A2	secretoglobin family 3A member 2	Lung	7.7	29_471	0.791
SFTPB	surfactant protein B	Lung	12.2	29_383	0.789
SCGB1A1	secretoglobin family 1A member 1	Lung	9.2	29_155	0.776
NAPSA	napsin A aspartic peptidase	Lung	8.8	30_291	0.774
STATH	statherin	Minor Salivary Gland	7.7	25_200	0.767
IDI2	isopentenyl-diphosphate delta isomerase 2	Muscle - Skeletal	8.0	30_161	0.916
RP11-451G4.2		Muscle - Skeletal	10.3	30_161	0.883
RP11-184I16.4		Muscle - Skeletal	7.2	30_161	0.881
MYF6	myogenic factor 6	Muscle - Skeletal	7.2	25_409	0.872
NEB	nebulin	Muscle - Skeletal	9.9	25_409	0.851
PPP1R27	protein phosphatase 1 regulatory subunit 27	Muscle - Skeletal	9.0	30_161	0.842
CACNG1	calcium voltage-gated channel auxiliary subunit gamma 1	Muscle - Skeletal	7.8	25_409	0.836
XIRP2	xin actin binding repeat containing 2	Muscle - Skeletal	8.0	18_102	0.828
RYR1	ryanodine receptor 1	Muscle - Skeletal	8.6	29_362	0.827
TMOD4	tropomodulin 4	Muscle - Skeletal	8.6	30_16	0.827
KLHL40	kelch like family member 40	Muscle - Skeletal	7.8	25_409	0.824
MYBPC2	myosin binding protein C2	Muscle - Skeletal	9.4	30_161	0.822
SMTNL1	smoothelin like 1	Muscle - Skeletal	7.2	30_362	0.820
TTN	titin	Muscle - Skeletal	8.7	18_136	0.810
MYH1	myosin heavy chain 1	Muscle - Skeletal	10.3	25_409	0.808
MYPN	myopalladin	Muscle - Skeletal	7.3	30_72	0.805
LMOD3	leiomodin 3	Muscle - Skeletal	7.4	18_136	0.802
MYLPF	myosin light chain, phosphorylatable, fast skeletal muscle	Muscle - Skeletal	10.9	30_161	0.798
LMOD2	leiomodin 2	Muscle - Skeletal	8.9	30_72	0.798
NRAP	nebulin related anchoring protein	Muscle - Skeletal	10.0	18_136	0.794
MYL1	myosin light chain 1	Muscle - Skeletal	11.2	30_161	0.789
ATP2A1	ATPase sarcoplasmic/endoplasmic reticulum Ca2+ transporting 1	Muscle - Skeletal	10.0	30_161	0.784
TNNC2	troponin C2, fast skeletal type	Muscle - Skeletal	12.5	30_161	0.782
MYL2	myosin light chain 2	Muscle - Skeletal	13.7	18_136	0.781

Gene	Gene name	Tissue with highest expression	Highest median tissue expression (log_2)	Best correlated feature	r
RP11-357D18.1		Muscle - Skeletal	7.3	27_117	0.779
AMPD1	adenosine monophosphate deaminase 1	Muscle - Skeletal	8.5	30_16	0.779
MYH2	myosin heavy chain 2	Muscle - Skeletal	9.4	30_505	0.779
MYLK2	myosin light chain kinase 2	Muscle - Skeletal	7.7	30_161	0.777
TNNT1	troponin T1, slow skeletal type	Muscle - Skeletal	12.5	18_272	0.776
TRIM63	tripartite motif containing 63	Muscle - Skeletal	9.6	18_136	0.776
TNNI1	troponin I1, slow skeletal type	Muscle - Skeletal	10.0	30_161	0.773
HFE2	hemojuvelin BMP co-receptor	Muscle - Skeletal	7.8	20_305	0.773
MB	myoglobin	Muscle - Skeletal	13.1	30_72	0.772
MYH7	myosin heavy chain 7	Muscle - Skeletal	12.4	18_136	0.770
RPL3L	ribosomal protein L3 like	Muscle - Skeletal	8.2	27_409	0.767
KLHL41	kelch like family member 41	Muscle - Skeletal	11.6	20_171	0.766
COX6A2	cytochrome c oxidase subunit 6A2	Muscle - Skeletal	11.5	30_72	0.759
SLN	sarcolipin	Muscle - Skeletal	10.9	30_72	0.758
ANP32B	acidic nuclear phosphoprotein 32 family member B	Nerve - Tibial	8.2	19_468	0.775
CNTF	ciliary neurotrophic factor	Nerve - Tibial	8.3	29_36	0.769
AHNAK	AHNAK nucleoprotein	Nerve - Tibial	10.0	14_162	0.754
MXRA8	matrix remodeling associated 8	Nerve - Tibial	8.5	19_468	0.751
RPL22	ribosomal protein L22	Ovary	8.9	19_468	0.779
ZFAS1	ZNFX1 antisense RNA 1	Ovary	8.8	19_468	0.775
EEF1A1	eukaryotic translation elongation factor 1 alpha 1	Ovary	12.3	12_164	0.774
RPL3	ribosomal protein L3	Ovary	12.2	12_164	0.765
HNRNPA1	heterogeneous nuclear ribonucleoprotein A1	Ovary	10.0	19_468	0.754
RPL15	ribosomal protein L15	Ovary	9.8	19_468	0.753
CLTA	clathrin light chain A	Ovary	7.5	19_321	0.752
PNLIPRP1	pancreatic lipase related protein 1	Pancreas	11.5	18_425	0.944
SERPINI2	serpin family I member 2	Pancreas	8.0	29_467	0.941
RBPJL	recombination signal binding protein for immunoglobulin kappa J region like	Pancreas	9.5	30_467	0.935
CTRC	chymotrypsin C	Pancreas	12.5	30_467	0.933
RP11-331F4.4		Pancreas	11.6	18_425	0.932
AMY2A	amylase alpha 2A	Pancreas	13.1	30_467	0.928
CELA2B	chymotrypsin like elastase 2B	Pancreas	11.3	30_467	0.917
INS	insulin	Pancreas	10.8	18_158	0.913
SYCN	syncollin	Pancreas	13.8	30_290	0.902
CELA2A	chymotrypsin like elastase 2A	Pancreas	12.6	23_110	0.895
CUZD1	CUB and zona pellucida like domains 1	Pancreas	8.9	22_324	0.895
CTRL	chymotrypsin like	Pancreas	10.8	20_292	0.893
CELP	carboxyl ester lipase pseudogene	Pancreas	8.0	23_324	0.893
CELA3B	chymotrypsin like elastase 3B	Pancreas	13.2	18_425	0.881
CEL	carboxyl ester lipase	Pancreas	14.0	23_324	0.866
CTRB1	chymotrypsinogen B1	Pancreas	14.3	18_158	0.861
PRSS3P2	PRSS3 pseudogene 2	Pancreas	13.4	18_123	0.861
PNLIPRP2	pancreatic lipase related protein 2 (gene/pseudogene)	Pancreas	12.6	18_123	0.851
CPA2	carboxypeptidase A2	Pancreas	13.7	18_123	0.850
PLA2G1B	phospholipase A2 group IB	Pancreas	14.4	18_158	0.846
CTRB2	chymotrypsinogen B2	Pancreas	14.3	18_158	0.845
PRSS3P1	PRSS3 pseudogene 1	Pancreas	13.5	18_123	0.844
PNLIP	pancreatic lipase	Pancreas	16.2	18_123	0.831
GP2	glycoprotein 2	Pancreas	13.6	18_483	0.828
CELA3A	chymotrypsin like elastase 3A	Pancreas	15.0	18_123	0.828
CPB1	carboxypeptidase B1	Pancreas	14.4	18_137	0.822
RP11-133O22.6		Pancreas	8.3	30_290	0.819
CPA1	carboxypeptidase A1	Pancreas	15.9	18_158	0.819

Gene	Gene name	Tissue with highest expression	Highest median tissue expression (*log*$_2$)	Best correlated feature	r
REG3G	regenerating family member 3 gamma	Pancreas	9.6	30_290	0.815
CLPS	colipase	Pancreas	16.2	18_123	0.808
RP6-149D17.1		Pancreas	7.8	30_467	0.790
ERP27	endoplasmic reticulum protein 27	Pancreas	8.6	18_377	0.780
PRSS1	serine protease 1	Pancreas	17.0	18_123	0.768
GCG	glucagon	Pancreas	7.8	23_188	0.767
AMY2B	amylase alpha 2B	Pancreas	10.6	22_246	0.754
FSHB	follicle stimulating hormone subunit beta	Pituitary	7.1	29_279	0.930
POU1F1	POU class 1 homeobox 1	Pituitary	7.3	29_279	0.913
GHRHR	growth hormone releasing hormone receptor	Pituitary	8.9	29_279	0.900
TSHB	thyroid stimulating hormone subunit beta	Pituitary	9.2	29_436	0.869
CGA	glycoprotein hormones, alpha polypeptide	Pituitary	13.3	30_429	0.852
LHB	luteinizing hormone subunit beta	Pituitary	12.0	30_429	0.844
OTOS	otospiralin	Pituitary	7.2	29_413	0.831
MYO15A	myosin XVA	Pituitary	7.2	29_436	0.803
PRL	prolactin	Pituitary	15.5	29_436	0.799
POMC	proopiomelanocortin	Pituitary	15.0	29_279	0.794
RP11-566J3.4		Pituitary	7.9	27_217	0.789
TGFBR3L	transforming growth factor beta receptor 3 like	Pituitary	8.0	29_436	0.787
ARHGAP36	Rho GTPase activating protein 36	Pituitary	8.4	29_429	0.786
MIR7-3HG	MIR7-3 host gene	Pituitary	11.3	29_279	0.763
GH1	growth hormone 1	Pituitary	16.8	30_429	0.763
COL22A1	collagen type XXII alpha 1 chain	Pituitary	7.2	30_436	0.753
KLK3	kallikrein related peptidase 3	Prostate	12.8	29_458	0.900
KLK2	kallikrein related peptidase 2	Prostate	11.5	29_193	0.898
KLK4	kallikrein related peptidase 4	Prostate	9.4	29_430	0.821
HOXB13	homeobox B13	Prostate	7.3	30_458	0.801
ACPP	acid phosphatase 3	Prostate	10.5	30_339	0.764
MSMB	microseminoprotein beta	Prostate	10.8	30_458	0.764
KRT77	keratin 77	Skin - Not Sun Exposed	8.6	18_78	0.884
DCD	dermcidin	Skin - Not Sun Exposed	11.4	22_265	0.848
MUCL1	mucin like 1	Skin - Not Sun Exposed	9.7	18_78	0.820
C1orf68	chromosome 1 open reading frame 68	Skin - Sun Exposed	9.6	18_78	0.884
FLG2	filaggrin family member 2	Skin - Sun Exposed	9.6	18_78	0.883
SERPINA12	serpin family A member 12	Skin - Sun Exposed	8.9	18_78	0.880
LCE2C	late cornified envelope 2C	Skin - Sun Exposed	8.2	18_78	0.878
LCE1A	late cornified envelope 1A	Skin - Sun Exposed	8.9	18_78	0.878
CDSN	corneodesmosin	Skin - Sun Exposed	8.3	18_78	0.875
LCE2B	late cornified envelope 2B	Skin - Sun Exposed	9.4	18_78	0.874
LCE1B	late cornified envelope 1B	Skin - Sun Exposed	8.0	18_78	0.874
LCE1C	late cornified envelope 1C	Skin - Sun Exposed	9.4	18_78	0.873
LCE6A	late cornified envelope 6A	Skin - Sun Exposed	7.6	18_78	0.870
LCE1F	late cornified envelope 1F	Skin - Sun Exposed	7.6	18_78	0.868
LCE5A	late cornified envelope 5A	Skin - Sun Exposed	7.3	18_78	0.864
LCE2D	late cornified envelope 2D	Skin - Sun Exposed	7.5	18_78	0.863
LOR	loricrin cornified envelope precursor protein	Skin - Sun Exposed	11.5	18_78	0.857
LCE2A	late cornified envelope 2A	Skin - Sun Exposed	7.2	18_78	0.856
DSC1	desmocollin 1	Skin - Sun Exposed	8.2	18_78	0.849
KRT2	keratin 2	Skin - Sun Exposed	12.8	18_78	0.840
FLG	filaggrin	Skin - Sun Exposed	9.0	18_78	0.835
SERPINB7	serpin family B member 7	Skin - Sun Exposed	7.4	18_78	0.830
KRT10	keratin 10	Skin - Sun Exposed	14.6	18_78	0.826
CASP14	caspase 14	Skin - Sun Exposed	9.4	18_78	0.820
WFDC5	WAP four-disulfide core domain 5	Skin - Sun Exposed	8.7	18_78	0.818
CCL27	C-C motif chemokine ligand 27	Skin - Sun Exposed	7.2	18_78	0.812
PSAPL1	prosaposin like 1	Skin - Sun Exposed	7.4	22_265	0.812

Gene	Gene name	Tissue with highest expression	Highest median tissue expression (log_2)	Best correlated feature	r
THEM5	thioesterase superfamily member 5	Skin - Sun Exposed	7.9	18_78	0.795
KPRP	keratinocyte proline rich protein	Skin - Sun Exposed	9.2	18_78	0.794
PSORS1C2	psoriasis susceptibility 1 candidate 2	Skin - Sun Exposed	7.7	18_78	0.781
CST6	cystatin E/M	Skin - Sun Exposed	9.6	20_329	0.773
ASPRV1	aspartic peptidase retroviral like 1	Skin - Sun Exposed	9.6	18_78	0.772
LY6G6C	lymphocyte antigen 6 family member G6C	Skin - Sun Exposed	9.5	25_227	0.757
ALOX12B	arachidonate 12-lipoxygenase, 12R type	Skin - Sun Exposed	7.4	25_227	0.757
SPINK4	serine peptidase inhibitor Kazal type 4	Small Intestine - Terminal Ileum	7.7	29_390	0.765
CD5L	CD5 molecule like	Spleen	9.2	22_385	0.936
NR5A1	nuclear receptor subfamily 5 group A member 1	Spleen	8.0	27_423	0.780
CD19	CD19 molecule	Spleen	7.9	27_389	0.759
GIF	cobalamin binding intrinsic factor	Stomach	10.8	29_287	0.877
PGA4	pepsinogen A4	Stomach	9.3	29_287	0.872
ATP4B	ATPase H+/K+ transporting subunit beta	Stomach	10.6	29_287	0.865
PGA5	pepsinogen A5	Stomach	8.8	29_287	0.864
PGA3	pepsinogen A3	Stomach	8.5	29_287	0.816
MUC5AC	mucin 5AC, oligomeric mucus/gel-forming	Stomach	10.5	29_287	0.811
TFF2	trefoil factor 2	Stomach	11.2	29_287	0.801
GKN1	gastrokine 1	Stomach	12.2	29_287	0.791
KCNE2	potassium voltage-gated channel subfamily E regulatory subunit 2	Stomach	8.3	29_287	0.782
DDX4	DEAD-box helicase 4	Testis	7.5	29_39	0.951
HDGFL1	HDGF like 1	Testis	7.5	29_39	0.951
DMRTB1	DMRT like family B with proline rich C-terminal 1	Testis	7.1	29_39	0.949
FAM71F1	family with sequence similarity 71 member F1	Testis	7.4	29_39	0.947
SHCBP1L	SHC binding and spindle associated 1 like	Testis	7.7	29_39	0.945
AC114783.1		Testis	7.0	29_73	0.945
CALR3	calreticulin 3	Testis	7.1	29_246	0.945
LYPD4	LY6/PLAUR domain containing 4	Testis	7.8	29_39	0.945
SPACA7	sperm acrosome associated 7	Testis	7.3	29_246	0.945
LINC00917	long intergenic non-protein coding RNA 917	Testis	7.7	29_246	0.944
ANKRD7	ankyrin repeat domain 7	Testis	9.6	29_39	0.944
PRED58		Testis	7.8	29_39	0.943
LYZL2	lysozyme like 2	Testis	7.1	29_73	0.943
IQCF2	IQ motif containing F2	Testis	7.5	29_246	0.943
LINC00202-2	family with sequence similarity 238 member B	Testis	7.6	29_246	0.942
GK2	glycerol kinase 2	Testis	7.1	29_73	0.942
TSPAN16	tetraspanin 16	Testis	7.3	29_246	0.942
CETN1	centrin 1	Testis	8.8	29_39	0.942
TEX33	testis expressed 33	Testis	7.2	29_246	0.942
ZPBP2	zona pellucida binding protein 2	Testis	7.2	29_39	0.941
RP11-71E19.5		Testis	7.1	29_246	0.941
RP11-404K5.2		Testis	7.0	29_39	0.940
PDCL2	phosducin like 2	Testis	7.0	29_168	0.940
DEFB123	defensin beta 123	Testis	7.7	29_39	0.940
TMCO2	transmembrane and coiled-coil domains 2	Testis	7.6	29_246	0.940
TEX37	testis expressed 37	Testis	7.3	29_246	0.940
KIF2B	kinesin family member 2B	Testis	7.3	29_73	0.940
SPERT	chibby family member 2	Testis	7.3	29_73	0.940
CCDC70	coiled-coil domain containing 70	Testis	8.3	29_246	0.940
PPP1R2P9	PPP1R2C family member C	Testis	7.3	29_73	0.939
ACTL9	actin like 9	Testis	8.2	29_246	0.939
SPEM1	spermatid maturation 1	Testis	7.5	29_246	0.939
ACTL7B	actin like 7B	Testis	8.3	29_246	0.938
ZNRF4	zinc and ring finger 4	Testis	8.4	29_246	0.938
ACSBG2	acyl-CoA synthetase bubblegum family member 2	Testis	8.0	29_39	0.938
C17orf74	SPEM family member 2	Testis	8.3	29_246	0.938

Gene	Gene name	Tissue with highest expression	Highest median tissue expression (log_2)	Best correlated feature	r
IRGC	immunity related GTPase cinema	Testis	8.5	29_39	0.937
SPATA19	spermatogenesis associated 19	Testis	8.0	29_168	0.937
TEX38	testis expressed 38	Testis	8.9	29_39	0.937
C10orf62	chromosome 10 open reading frame 62	Testis	9.3	29_39	0.937
ACTRT2	actin related protein T2	Testis	8.8	29_246	0.937
RNF151	ring finger protein 151	Testis	8.5	29_39	0.937
C17orf64	chromosome 17 open reading frame 64	Testis	7.9	29_39	0.937
RP11-314A5.3		Testis	7.6	29_246	0.937
C2orf53	proline rich 30	Testis	8.3	29_73	0.936
PGK2	phosphoglycerate kinase 2	Testis	8.3	29_39	0.936
UBQLN3	ubiquilin 3	Testis	8.4	29_246	0.935
BOD1L2	biorientation of chromosomes in cell division 1 like 2	Testis	9.2	29_39	0.935
FNDC8	fibronectin type III domain containing 8	Testis	7.2	29_246	0.934
SPATA42	spermatogenesis associated 42	Testis	7.8	29_246	0.934
HMGB4	high mobility group box 4	Testis	9.1	29_73	0.934
DEFB119	defensin beta 119	Testis	8.2	29_39	0.933
FSCN3	fascin actin-bundling protein 3	Testis	7.2	29_246	0.933
AKAP4	A-kinase anchoring protein 4	Testis	8.8	29_246	0.933
CCDC54	coiled-coil domain containing 54	Testis	8.2	29_39	0.933
WBSCR28	transmembrane protein 270	Testis	7.8	29_246	0.932
C2orf57	testis expressed 44	Testis	8.5	29_246	0.932
CST9L	cystatin 9 like	Testis	7.0	29_39	0.932
C16orf82	chromosome 16 open reading frame 82	Testis	9.3	29_39	0.932
FAM187B	family with sequence similarity 187 member B	Testis	7.0	29_246	0.932
C17orf50	chromosome 17 open reading frame 50	Testis	7.7	29_39	0.931
C1orf234	testis expressed 46	Testis	7.0	29_73	0.931
ACTL7A	actin like 7A	Testis	9.1	29_73	0.931
SEPT12	septin 12	Testis	7.8	29_39	0.930
SPATA8	spermatogenesis associated 8	Testis	9.6	29_39	0.930
TCP11	t-complex 11	Testis	8.7	29_258	0.930
TUBA3C	tubulin alpha 3c	Testis	10.0	29_39	0.929
GTSF1L	gametocyte specific factor 1 like	Testis	8.9	29_246	0.929
ODF1	outer dense fiber of sperm tails 1	Testis	9.6	29_39	0.929
SMCP	sperm mitochondria associated cysteine rich protein	Testis	10.7	29_39	0.928
GSG1	germ cell associated 1	Testis	8.5	29_39	0.928
IQCF1	IQ motif containing F1	Testis	7.8	29_246	0.926
PRM3	protamine 3	Testis	7.0	29_246	0.926
LINC00238	coiled-coil domain containing 196	Testis	7.2	29_246	0.924
SPATA22	spermatogenesis associated 22	Testis	7.2	29_39	0.923
TDRG1	testis development related 1	Testis	7.0	29_39	0.923
CDIPT-AS1	CDIP transferase opposite strand, pseudogene	Testis	7.9	29_39	0.922
LDHC	lactate dehydrogenase C	Testis	8.4	29_39	0.921
GAPDHS	glyceraldehyde-3-phosphate dehydrogenase, spermatogenic	Testis	8.6	29_73	0.921
FATE1	fetal and adult testis expressed 1	Testis	8.8	29_39	0.920
LELP1	late cornified envelope like proline rich 1	Testis	10.8	29_73	0.917
SYCP3	synaptonemal complex protein 3	Testis	7.2	29_39	0.917
AC010982.1		Testis	8.1	29_258	0.916
C6orf99	chromosome 6 open reading frame 99	Testis	7.3	29_39	0.916
CABS1	calcium binding protein, spermatid associated 1	Testis	7.9	29_73	0.915
EPPIN	epididymal peptidase inhibitor	Testis	7.0	29_39	0.915
DKKL1	dickkopf like acrosomal protein 1	Testis	9.3	29_39	0.914
H1FNT	H1.7 linker histone	Testis	9.0	29_39	0.913
TSACC	TSSK6 activating cochaperone	Testis	9.8	29_168	0.912
C20orf141	chromosome 20 open reading frame 141	Testis	7.9	29_73	0.911
RPL10L	ribosomal protein L10 like	Testis	7.5	29_246	0.908
SYCE3	synaptonemal complex central element protein 3	Testis	7.9	29_39	0.906

Gene	Gene name	Tissue with highest expression	Highest median tissue expression (*log*$_2$)	Best correlated feature	r
CAPZA3	capping actin protein of muscle Z-line subunit alpha 3	Testis	8.7	29_258	0.906
GTSF1	gametocyte specific factor 1	Testis	7.0	29_246	0.905
CCDC42	coiled-coil domain containing 42	Testis	7.0	29_73	0.903
TXNDC2	thioredoxin domain containing 2	Testis	7.1	29_246	0.898
UBL4B	ubiquitin like 4B	Testis	8.1	29_39	0.897
TKTL1	transketolase like 1	Testis	7.7	29_39	0.895
SPACA3	sperm acrosome associated 3	Testis	7.7	29_39	0.895
RBAKDN	RBAK downstream neighbor	Testis	10.1	29_258	0.889
TPPP2	tubulin polymerization promoting protein family member 2	Testis	8.2	29_73	0.888
FAM209A	family with sequence similarity 209 member A	Testis	7.0	29_246	0.880
AKAP3	A-kinase anchoring protein 3	Testis	7.3	29_39	0.880
SPTY2D1-AS1	SPTY2D1 opposite strand	Testis	7.0	29_73	0.878
MRVI1-AS1	IRAG1 antisense RNA 1	Testis	7.2	29_246	0.878
TEX40	catsper channel auxiliary subunit zeta	Testis	10.0	29_39	0.877
PFN4	profilin family member 4	Testis	7.5	29_39	0.876
PRND	prion like protein doppel	Testis	7.1	29_39	0.876
TMEM31	transmembrane protein 31	Testis	7.0	29_39	0.876
TNP1	transition protein 1	Testis	13.1	29_39	0.874
TSSK6	testis specific serine kinase 6	Testis	8.2	29_246	0.869
FBXO24	F-box protein 24	Testis	7.1	29_39	0.868
C20orf195	fibronectin type III domain containing 11	Testis	8.0	29_39	0.867
PHF7	PHD finger protein 7	Testis	10.9	29_258	0.867
TEX101	testis expressed 101	Testis	7.7	29_168	0.866
MAEL	maelstrom spermatogenic transposon silencer	Testis	7.3	29_39	0.864
TCP10L	t-complex 10 like	Testis	8.1	29_39	0.864
LEMD1	LEM domain containing 1	Testis	7.3	29_39	0.863
CCIN	calicin	Testis	7.3	29_73	0.862
PRAME	PRAME nuclear receptor transcriptional regulator	Testis	7.3	30_39	0.860
FAM209B	family with sequence similarity 209 member B	Testis	7.4	29_246	0.858
CMTM2	CKLF like MARVEL transmembrane domain containing 2	Testis	10.3	29_246	0.851
SMKR1	small lysine rich protein 1	Testis	8.2	30_39	0.850
PRM1	protamine 1	Testis	14.3	29_73	0.850
PRM2	protamine 2	Testis	14.3	29_73	0.849
GGN	gametogenetin	Testis	7.5	29_39	0.845
DYNLRB2	dynein light chain roadblock-type 2	Testis	7.4	29_39	0.845
ROPN1L	rhophilin associated tail protein 1 like	Testis	9.0	29_39	0.844
TSKS	testis specific serine kinase substrate	Testis	8.3	29_258	0.842
CCDC96	coiled-coil domain containing 96	Testis	7.1	29_39	0.842
CLPB	caseinolytic mitochondrial matrix peptidase chaperone subunit B	Testis	8.2	29_372	0.841
CABYR	calcium binding tyrosine phosphorylation regulated	Testis	8.0	29_39	0.840
RP11-758P17.3		Testis	7.5	29_285	0.832
ZMYND10	zinc finger MYND-type containing 10	Testis	8.5	30_39	0.829
LINC00467	long intergenic non-protein coding RNA 467	Testis	9.5	30_14	0.827
CRISP2	cysteine rich secretory protein 2	Testis	10.0	29_168	0.825
AC004381.6		Testis	8.8	29_168	0.823
ACTL10	actin like 10	Testis	7.2	29_73	0.817
FAM71E1	family with sequence similarity 71 member E1	Testis	8.3	30_39	0.811
CCDC11	cilia and flagella associated protein 53	Testis	7.4	30_39	0.810
GS1-259H13.2		Testis	7.7	30_258	0.809
RP11-496H1.2		Testis	7.4	29_39	0.808
INSL3	insulin like 3	Testis	8.9	29_39	0.802
ADAD2	adenosine deaminase domain containing 2	Testis	7.5	29_285	0.799
ACRBP	acrosin binding protein	Testis	9.3	30_246	0.798
ABHD1	abhydrolase domain containing 1	Testis	7.1	29_39	0.789
COX6B2	cytochrome c oxidase subunit 6B2	Testis	7.3	29_258	0.786

Gene	Gene name	Tissue with highest expression	Highest median tissue expression (log_2)	Best correlated feature	r
SPACA4	sperm acrosome associated 4	Testis	7.9	30_246	0.785
PHOSPHO1	phosphoethanolamine/phosphocholine phosphatase 1	Testis	7.3	29_246	0.782
C1orf194	chromosome 1 open reading frame 194	Testis	8.3	30_39	0.778
SPATA24	spermatogenesis associated 24	Testis	7.5	29_73	0.778
CCT6B	chaperonin containing TCP1 subunit 6B	Testis	7.6	29_258	0.777
SOCS7	suppressor of cytokine signaling 7	Testis	7.6	29_379	0.776
ERICH2	glutamate rich 2	Testis	8.5	29_39	0.773
SPINK2	serine peptidase inhibitor Kazal type 2	Testis	9.2	29_73	0.773
SYCE1	synaptonemal complex central element protein 1	Testis	7.6	30_115	0.773
PCSK4	proprotein convertase subtilisin/kexin type 4	Testis	8.2	30_39	0.773
PRSS21	serine protease 21	Testis	7.0	30_168	0.773
TUBA3E	tubulin alpha 3e	Testis	8.5	30_55	0.765
ACR	acrosin	Testis	7.2	29_246	0.761
MIR202	microRNA 202	Testis	9.6	29_240	0.757
PROCA1	protein interacting with cyclin A1	Testis	8.0	29_39	0.756
ZDHHC19	zinc finger DHHC-type palmitoyltransferase 19	Testis	7.1	29_246	0.755
LRWD1	leucine rich repeats and WD repeat domain containing 1	Testis	9.4	30_246	0.754
TUBA3D	tubulin alpha 3d	Testis	8.7	30_73	0.752
CTD-2182N23.1		Thyroid	9.1	29_93	0.937
TSHR	thyroid stimulating hormone receptor	Thyroid	7.3	30_93	0.907
SLC26A7	solute carrier family 26 member 7	Thyroid	8.8	30_499	0.888
NKX2-1	NK2 homeobox 1	Thyroid	8.5	29_202	0.870
TG	thyroglobulin	Thyroid	12.3	30_93	0.869
SFTA3	surfactant associated 3	Thyroid	7.4	27_346	0.842
IYD	iodotyrosine deiodinase	Thyroid	8.5	29_93	0.836
SLC26A4-AS1	SLC26A4 antisense RNA 1	Thyroid	7.1	30_217	0.806
PAX8	paired box 8	Thyroid	10.4	29_499	0.802
FOXE1	forkhead box E1	Thyroid	7.5	27_376	0.778
PCED1A	PC-esterase domain containing 1A	Thyroid	7.5	26_506	0.751
TRIP6	thyroid hormone receptor interactor 6	Uterus	7.6	12_209	0.762
