# Supplementary material for: Identifying transcriptomic correlates of histology using deep learning
Source: PLoS One. 2020 Nov 25;15(11):e0242858. doi: 10.1371/journal.pone.0242858 (PMC7688140; doi:10.1371/journal.pone.0242858)
Supplement: S3 Table — Genes with Gene Ontology ‘developmental process’ or ‘transcription regulator activity’ annotations are grouped w.r.t. tissues and ordered by correlation—for each gene we only show the best correlated feature, in the form [layer]_[channel] (for the architecture VGG16 and the test dataset; correlation threshold = 0.75, log2 gene expression threshold = 7). Genes with ‘transcription regulator activity’ are shown in red. (PDF) [file pone.0242858.s006.pdf]

S3 Table. Developmental and transcription regulation genes correlated with visual features. Genes with Gene Ontology '*developmental process*' or '*transcription regulator activity*' annotations are grouped w.r.t. tissues and ordered by correlation - for each gene we only show the best correlated feature, in the form [layer]_[channel] (for the architecture VGG16 and the test dataset; correlation threshold=0.75, log_2 gene expression threshold=7). Genes with '*transcription regulator activity*' are shown in red.

Gene	Gene name	GO	Tissue with highest expression	Highest median tissue expression (log_2)	Best correlated feature	r
FABP4	Fatty acid-binding protein, adipocyte	white fat cell differentiation; brown fat cell differentiation	Adipose - Visceral (Omentum)	12.6	1_14	0.767
CYP17A1	Steroid 17-alpha-hydroxylase/17,20 lyase	sex differentiation	Adrenal Gland	12.4	20_467	0.805
HSD3B2	3 beta-hydroxysteroid dehydrogenase/Delta 5-->4-isomerase type 2	hippocampus development	Adrenal Gland	11.2	29_123	0.791
STAR	Steroidogenic acute regulatory protein, mitochondrial	brain development	Adrenal Gland	12.5	29_132	0.770
TPM4	Tropomyosin alpha-4 chain	osteoblast differentiation	Artery - Aorta	9.1	19_468	0.767
S100A6	Protein S100-A6	axonogenesis	Artery - Aorta	11.6	19_162	0.760
YAP1	Transcriptional coactivator YAP1	vasculogenesis	Artery - Aorta	7.6	19_162	0.758
MARVELD1	MARVEL domain-containing protein 1	myelination	Artery - Tibial	7.8	19_162	0.764
GNG12	Guanine nucleotide-binding protein G(I)/G(S)/G(O) subunit gamma-12	cerebral cortex development	Artery - Tibial	7.1	12_209	0.755
ZIC4	Zinc finger protein ZIC 4	central nervous system development	Brain - Cerebellum	7.0	29_447	0.922
NEUROD2	Neurogenic differentiation factor 2	cerebellar cortex development; positive regulation of neuron differentiation	Brain - Cerebellum	7.2	29_463	0.915
NEUROD1	Neurogenic differentiation factor 1	cerebellum development; neurogenesis	Brain - Cerebellum	7.8	29_479	0.873
CRTAM	Cytotoxic and regulatory T-cell molecule	regulation of T cell differentiation	Brain - Cerebellum	7.2	29_479	0.868
ZIC2	Zinc finger protein ZIC 2	brain development; cell differentiation	Brain - Cerebellum	7.9	29_463	0.854
SLC12A5	Solute carrier family 12 member 5	dendritic spine development; multicellular organism growth	Brain - Cerebellum	7.5	27_140	0.850
ZIC1	Zinc finger protein ZIC 1	brain development	Brain - Cerebellum	8.1	29_479	0.844
ELAVL3	ELAV-like protein 3	nervous system development; cell differentiation	Brain - Cerebellum	8.0	29_463	0.834
PVALB	Parvalbumin alpha	cochlea development	Brain - Cerebellum	9.1	29_463	0.781
HPCAL4	Hippocalcin-like protein 4	central nervous system development	Brain - Cerebellum	7.5	29_463	0.781
CPLX2	Complexin-2	nervous system development; cell differentiation	Brain - Cerebellum	8.7	27_140	0.762
SPOCK2	Testican-2	synapse assembly; regulation of cell differentiation	Brain - Cerebellum	8.6	27_148	0.761
SLC1A2	Excitatory amino acid transporter 2	telencephalon development; multicellular organism growth	Brain - Cortex	8.7	18_8	0.844
GRIN1	Glutamate receptor ionotropic, NMDA 1	cerebral cortex development	Brain - Cortex	7.9	18_8	0.812
HPCA	Neuron-specific calcium-binding protein hippocalcin	brain development	Brain - Cortex	7.5	29_485	0.811
PACSIN1	Protein kinase C and casein kinase substrate in neurons protein 1	neuron projection morphogenesis; positive regulation of dendrite development	Brain - Cortex	8.5	29_463	0.810
SLC17A7	Vesicular glutamate transporter 1	brain development; synaptic vesicle lumen acidification	Brain - Cortex	9.3	18_8	0.799
CAMK2A	Calcium/calmodulin-dependent protein kinase type II subunit alpha	dendritic spine development; regulation of neuron migration	Brain - Cortex	9.2	23_417	0.786
DDN	Dendrin	DNA-binding transcription factor activity, RNA polymerase II-specific	Brain - Cortex	7.9	25_478	0.775
CEND1	Cell cycle exit and neuronal differentiation protein 1	neuron differentiation	Brain - Cortex	8.6	29_485	0.755
KIF5A	Kinesin heavy chain isoform 5A	axon guidance	Brain - Cortex	10.0	29_485	0.754
NBL1	Neuroblastoma suppressor of tumorigenicity 1	animal organ morphogenesis	Cervix - Ectocervix	9.6	19_162	0.758
CRTAP	Cartilage-associated protein	spermatogenesis	Cervix - Ectocervix	8.1	12_223	0.756
HMGB1	High mobility group protein B1	developmental process	Cervix - Ectocervix	7.4	19_468	0.753
PIAS3	E3 SUMO-protein ligase PIAS3	transcription coregulator activity	Cervix - Endocervix	7.0	19_468	0.764

Gene	Gene name	GO	Tissue with highest expression	Highest median tissue expression (*log*$_2$)	Best correlated feature	r
SPIN1	Spindlin-1	multicellular organism development	Cervix - Endocervix	7.2	19_333	0.752
PLXNB2	Plexin-B2	brain development	Cervix - Endocervix	8.0	12_164	0.750
NKX2-5	Homeobox protein Nkx-2.5	atrial cardiac muscle cell development	Heart - Atrial Appendage	7.0	30_214	0.903
BMP10	Bone morphogenetic protein 10	atrial cardiac muscle tissue morphogenesis	Heart - Atrial Appendage	8.3	29_313	0.855
NPPA	Natriuretic peptides A	cell growth involved in cardiac muscle cell development	Heart - Atrial Appendage	14.9	29_481	0.777
NMRK2	Nicotinamide riboside kinase 2	negative regulation of myoblast differentiation	Heart - Atrial Appendage	9.8	30_72	0.751
MYBPC3	Myosin-binding protein C, cardiac-type	heart morphogenesis; ventricular cardiac muscle tissue morphogenesis	Heart - Left Ventricle	10.8	23_270	0.783
TNNI3	Troponin I, cardiac muscle	heart development; ventricular cardiac muscle tissue morphogenesis	Heart - Left Ventricle	12.0	25_28	0.780
TNNT2	Troponin T, cardiac muscle	ventricular cardiac muscle tissue morphogenesis; sarcomere organization	Heart - Left Ventricle	11.6	25_31	0.759
CSRP3	Cysteine and glycine-rich protein 3	cardiac muscle tissue development; cardiac myofibril assembly	Heart - Left Ventricle	9.4	30_72	0.755
AQP2	Aquaporin-2	metanephric collecting duct development	Kidney - Cortex	7.6	29_280	0.890
UMOD	Uromodulin	metanephric ascending thin limb development; metanephric distal convoluted tubule development	Kidney - Cortex	7.4	27_274	0.874
PLG	Plasminogen	tissue regeneration	Liver	8.7	29_234	0.959
APOA5	Apolipoprotein A-V	tissue regeneration	Liver	8.8	29_192	0.954
SERPINC1	Antithrombin-III	lactation	Liver	10.1	29_192	0.952
HRG	Histidine-rich glycoprotein	positive regulation of blood vessel remodeling	Liver	9.2	29_234	0.952
F2	Prothrombin	multicellular organism development; regulation of cell shape	Liver	9.4	29_192	0.949
AHSG	Alpha-2-HS-glycoprotein	skeletal system development	Liver	10.7	29_192	0.946
APCS	Serum amyloid P-component	negative regulation of monocyte differentiation	Liver	10.7	29_192	0.942
BAAT	Bile acid-CoA:amino acid N-acyltransferase	liver development; animal organ regeneration	Liver	7.7	29_192	0.938
ANGPTL3	Angiopoietin-related protein 3	artery morphogenesis	Liver	7.3	29_234	0.934
APOA2	Apolipoprotein A-II	animal organ regeneration	Liver	12.2	29_192	0.931
CYP4A11	Cytochrome P450 4A11	kidney development	Liver	8.3	29_234	0.924
CPB2	Carboxypeptidase B2	liver regeneration; negative regulation of hepatocyte proliferation	Liver	8.5	29_192	0.922
PROC	Vitamin K-dependent protein C	positive regulation of establishment of endothelial barrier	Liver	7.9	29_234	0.919
APOH	Beta-2-glycoprotein 1	negative regulation of angiogenesis	Liver	11.7	29_234	0.919
FGB	Fibrinogen beta chain	positive regulation of substrate adhesion-dependent cell spreading	Liver	12.6	29_192	0.896
FGL1	Fibrinogen-like protein 1	hepatocyte proliferation	Liver	10.2	18_231	0.879
G6PC	Glucose-6-phosphatase	multicellular organism growth	Liver	7.1	29_234	0.872
FGA	Fibrinogen alpha chain	positive regulation of substrate adhesion-dependent cell spreading	Liver	12.2	30_192	0.858
ASGR2	Asialoglycoprotein receptor 2	bone mineralization	Liver	8.8	30_192	0.854
CRP	C-reactive protein	negative regulation of macrophage derived foam cell differentiation	Liver	12.6	29_192	0.852
VTN	Vitronectin	liver regeneration; endodermal cell differentiation	Liver	11.2	18_279	0.851
FGG	Fibrinogen gamma chain	positive regulation of substrate adhesion-dependent cell spreading	Liver	12.2	30_192	0.829
IGFBP1	Insulin-like growth factor-binding protein 1	tissue regeneration	Liver	7.2	29_75	0.802
APOB	Apolipoprotein B-100	post-embryonic development	Liver	8.5	30_438	0.784
CREB3L3	Cyclic AMP-responsive element-binding protein 3-like protein 3	DNA-binding transcription factor activity, RNA polymerase II-specific	Liver	8.2	20_481	0.777
CPS1	Carbamoyl-phosphate synthase [ammonia], mitochondrial	hepatocyte differentiation; midgut development	Liver	8.6	29_192	0.775
ARG1	Arginase-1	liver development	Liver	9.0	20_194	0.768

Gene	Gene name	GO	Tissue with highest expression	Highest median tissue expression (log_2)	Best correlated feature	r
SFTPB	Pulmonary surfactant-associated protein B	animal organ morphogenesis	Lung	12.2	29_383	0.789
SCGB1A1	Uteroglobin	embryo implantation	Lung	9.2	29_155	0.776
STATH	Statherin	hepatocyte differentiation; midgut development	Minor Salivary Gland	7.7	25_200	0.767
MYF6	Myogenic factor 6	skeletal muscle tissue development; muscle cell fate commitment	Muscle - Skeletal	7.2	25_409	0.872
NEB	Nebulin	somatic muscle development; muscle fiber development	Muscle - Skeletal	9.9	25_409	0.851
XIRP2	Xin actin-binding repeat-containing protein 2	cardiac muscle tissue morphogenesis	Muscle - Skeletal	8.0	18_102	0.828
RYR1	Ryanodine receptor 1	skeletal muscle fiber development	Muscle - Skeletal	8.6	29_362	0.827
TMOD4	Tropomodulin-4	myofibril assembly	Muscle - Skeletal	8.6	30_16	0.827
KLHL40	Kelch-like protein 40	skeletal muscle fiber development; skeletal muscle fiber differentiation	Muscle - Skeletal	7.8	25_409	0.824
SMTNL1	Smoothelin-like protein 1	muscle organ morphogenesis	Muscle - Skeletal	7.2	30_362	0.820
TTN	Titin	skeletal muscle thin filament assembly; skeletal muscle myosin thick filament assembly; sarcomere organization	Muscle - Skeletal	8.7	18_136	0.810
MYPN	Myopalladin	sarcomere organization	Muscle - Skeletal	7.3	30_72	0.805
LMOD3	Leiomodin-3	skeletal muscle fiber development; myofibril assembly	Muscle - Skeletal	7.4	18_136	0.802
MYLPF	Myosin regulatory light chain 2, skeletal muscle isoform	skeletal muscle tissue development	Muscle - Skeletal	10.9	30_161	0.798
LMOD2	Leiomodin-2	sarcomere organization; myofibril assembly	Muscle - Skeletal	8.9	30_72	0.798
NRAP	Nebulin-related-anchoring protein	muscle fiber development	Muscle - Skeletal	10.0	18_136	0.794
MYL2	Myosin regulatory light chain 2, ventricular/cardiac muscle isoform	muscle cell fate specification; muscle fiber development	Muscle - Skeletal	13.7	18_136	0.781
MYLK2	Myosin light chain kinase 2, skeletal/cardiac muscle	skeletal muscle satellite cell differentiation; skeletal muscle cell differentiation	Muscle - Skeletal	7.7	30_161	0.777
TNNT1	Troponin T, slow skeletal muscle	sarcomere organization	Muscle - Skeletal	12.5	18_272	0.776
TNNI1	Troponin I, slow skeletal muscle	ventricular cardiac muscle tissue morphogenesis	Muscle - Skeletal	10.0	30_161	0.773
MB	Myoglobin	heart development	Muscle - Skeletal	13.1	30_72	0.772
MYH7	Myosin-7	adult heart development; ventricular cardiac muscle tissue morphogenesis	Muscle - Skeletal	12.4	18_136	0.770
KLHL41	Kelch-like protein 41	skeletal muscle fiber development; myofibril assembly	Muscle - Skeletal	11.6	20_171	0.766
ANP32B	Acidic leucine-rich nuclear phosphoprotein 32 family member B	negative regulation of cell differentiation	Nerve - Tibial	8.2	19_468	0.775
CNTF	Ciliary neurotrophic factor	neuron development; positive regulation of axon regeneration	Nerve - Tibial	8.3	29_36	0.769
MXRA8	Matrix remodeling-associated protein 8	establishment of glial blood-brain barrier	Nerve - Tibial	8.5	19_468	0.751
RBPJL	Recombining binding protein suppressor of hairless-like protein	DNA-binding transcription factor activity, RNA polymerase II-specific	Pancreas	9.5	30_467	0.935
INS	Insulin	positive regulation of cell differentiation	Pancreas	10.8	18_158	0.913
CELA2A	Chymotrypsin-like elastase family member 2A	cornification	Pancreas	12.6	23_110	0.895
CEL	Bile salt-activated lipase	presynaptic membrane assembly	Pancreas	14.0	23_324	0.866
REG3G	Regenerating islet-derived protein 3-gamma	negative regulation of keratinocyte differentiation	Pancreas	9.6	30_290	0.815
FSHB	Follitropin subunit beta	follicle-stimulating hormone signaling pathway	Pituitary	7.1	29_279	0.930
POU1F1	Pituitary-specific positive transcription factor 1	somatotropin secreting cell development	Pituitary	7.3	29_279	0.913
GHRHR	Growth hormone-releasing hormone receptor	somatotropin secreting cell development	Pituitary	8.9	29_279	0.900
TSHB	Thyrotropin subunit beta	anatomical structure morphogenesis	Pituitary	9.2	29_436	0.869
LHB	Lutropin subunit beta	male gonad development	Pituitary	12.0	30_429	0.844
MYO15A	Unconventional myosin-XV	inner ear morphogenesis	Pituitary	7.2	29_436	0.803
PRL	Prolactin	positive regulation of lactation	Pituitary	15.5	29_436	0.799

Gene	Gene name	GO	Tissue with highest expression	Highest median tissue expression (log_2)	Best correlated feature	r
TGFBR3L	Transforming growth factor-beta receptor type 3-like protein	epithelial to mesenchymal transition	Pituitary	8.0	29_436	0.787
GH1	Somatotropin	positive regulation of multicellular organism growth	Pituitary	16.8	30_429	0.763
COL22A1	Collagen alpha-1(XXII) chain	endothelial cell morphogenesis	Pituitary	7.2	30_436	0.753
KLK3	Prostate-specific antigen	negative regulation of angiogenesis	Prostate	12.8	29_458	0.900
KLK4	Kallikrein-4	biomineral tissue development	Prostate	9.4	29_430	0.821
HOXB13	Homeobox protein Hox-B13	prostate epithelial cord arborization involved in prostate glandular acinus morphogenesis; epithelial cell maturation involved in prostate gland development	Prostate	7.3	30_458	0.801
KRT77	Keratin, type II cytoskeletal 1b	keratinization; cornification	Skin - Not Sun Exposed	8.6	18_78	0.884
FLG2	Filaggrin-2	epidermis morphogenesis; establishment of skin barrier	Skin - Sun Exposed	9.6	18_78	0.883
LCE2C	Late cornified envelope protein 2C	keratinization	Skin - Sun Exposed	8.2	18_78	0.878
LCE1A	Late cornified envelope protein 1A	cornification	Skin - Sun Exposed	8.9	18_78	0.878
CDSN	Corneodesmosin	epidermis development; keratinocyte differentiation	Skin - Sun Exposed	8.3	18_78	0.875
LCE2B	Late cornified envelope protein 2B	epidermis development; keratinization	Skin - Sun Exposed	9.4	18_78	0.874
LCE1B	Late cornified envelope protein 1B	keratinization	Skin - Sun Exposed	8.0	18_78	0.874
LCE1C	Late cornified envelope protein 1C	keratinization	Skin - Sun Exposed	9.4	18_78	0.873
LCE6A	Late cornified envelope protein 6A	keratinization	Skin - Sun Exposed	7.6	18_78	0.870
LCE1F	Late cornified envelope protein 1F	keratinization	Skin - Sun Exposed	7.6	18_78	0.868
LCE5A	Late cornified envelope protein 5A	keratinization	Skin - Sun Exposed	7.3	18_78	0.864
LCE2D	Late cornified envelope protein 2D	keratinization	Skin - Sun Exposed	7.5	18_78	0.863
LCE2A	Late cornified envelope protein 2A	keratinization	Skin - Sun Exposed	7.2	18_78	0.856
DSC1	Desmocollin-1	keratinization; cornification	Skin - Sun Exposed	8.2	18_78	0.849
KRT2	Keratin, type II cytoskeletal 2 epidermal	keratinocyte development; epidermis development	Skin - Sun Exposed	12.8	18_78	0.840
FLG	Filaggrin	keratinocyte differentiation; establishment of skin barrier	Skin - Sun Exposed	9.0	18_78	0.835
SERPINB7	Serpin B7	positive regulation of glomerular mesangial cell proliferation	Skin - Sun Exposed	7.4	18_78	0.830
KRT10	Keratin, type I cytoskeletal 10	keratinocyte differentiation; positive regulation of epidermis development	Skin - Sun Exposed	14.6	18_78	0.826
CASP14	Caspase-14	epidermis development; keratinization	Skin - Sun Exposed	9.4	18_78	0.820
PSAPL1	Proactivator polypeptide-like 1	epithelial cell differentiation involved in prostate gland development	Skin - Sun Exposed	7.4	22_265	0.812
CST6	Cystatin-M	epidermis development; anatomical structure morphogenesis	Skin - Sun Exposed	9.6	20_329	0.773
ASPRV1	Retroviral-like aspartic protease 1	skin development	Skin - Sun Exposed	9.6	18_78	0.772
ALOX12B	Arachidonate 12-lipoxygenase, 12R-type	establishment of skin barrier	Skin - Sun Exposed	7.4	25_227	0.757
NR5A1	Steroidogenic factor 1	DNA-binding transcription factor activity, RNA polymerase II-specific; adrenal gland development	Spleen; Adrenal Gland	8.0	27_423	0.780
CD19	B-lymphocyte antigen CD19	B-1 B cell differentiation	Spleen	7.9	27_389	0.759
KCNE2	Potassium voltage-gated channel subfamily E member 2	tongue development	Stomach	8.3	29_287	0.782
DDX4	Probable ATP-dependent RNA helicase DDX4	spermatogenesis; cell differentiation	Testis	7.5	29_39	0.951
DMRTB1	Doublesex- and mab-3-related transcription factor B1	DNA-binding transcription factor activity, RNA polymerase II-specific	Testis	7.1	29_39	0.949
SHCBP1L	Testicular spindle-associated protein SHCBP1L	spermatogenesis; cell differentiation	Testis	7.7	29_39	0.945
CALR3	Calreticulin-3	spermatogenesis; cell differentiation	Testis	7.1	29_246	0.945
ZPBP2	Zona pellucida-binding protein 2	acrosome assembly	Testis	7.2	29_39	0.941
SPEM1	Spermatid maturation protein 1	sperm individualization; spermatogenesis	Testis	7.5	29_246	0.939
ACSBG2	Long-chain-fatty-acid--CoA ligase ACSBG2	spermatogenesis; cell differentiation	Testis	8.0	29_39	0.938
SPATA19	Spermatogenesis-associated protein 19, mitochondrial	spermatogenesis; cell differentiation	Testis	8.0	29_168	0.937

Gene	Gene name	GO	Tissue with highest expression	Highest median tissue expression (*log*$_2$)	Best correlated feature	r
RNF151	RING finger protein 151	spermatogenesis; cell differentiation	Testis	8.5	29_39	0.937
FSCN3	Fascin-3	spermatid development	Testis	7.2	29_246	0.933
TCP11	T-complex protein 11 homolog	spermatogenesis; cell differentiation	Testis	8.7	29_258	0.930
ODF1	Outer dense fiber protein 1	spermatogenesis; cell differentiation	Testis	9.6	29_39	0.929
IQCF1	IQ domain-containing protein F1	positive regulation of flagellated sperm motility involved in capacitation	Testis	7.8	29_246	0.926
PRM3	Protamine-3	spermatogenesis; cell differentiation	Testis	7.0	29_246	0.926
TDRG1	Testis development-related protein 1	multicellular organism development	Testis	7.0	29_39	0.923
SYCP3	Synaptonemal complex protein 3	spermatogenesis; spermatid development	Testis	7.2	29_39	0.917
CABS1	Calcium-binding and spermatid-specific protein 1	spermatogenesis	Testis	7.9	29_73	0.915
DKKL1	Dickkopf-like protein 1	anatomical structure morphogenesis	Testis	9.3	29_39	0.914
RPL10L	60S ribosomal protein L10-like	spermatogenesis	Testis	7.5	29_246	0.908
SYCE3	Synaptonemal complex central element protein 3	spermatogenesis	Testis	7.9	29_39	0.906
CAPZA3	F-actin-capping protein subunit alpha-3	spermatid development	Testis	8.7	29_258	0.906
GTSF1	Gametocyte-specific factor 1	spermatogenesis; cell differentiation	Testis	7.0	29_246	0.905
CCDC42	Coiled-coil domain-containing protein 42	spermatid development	Testis	7.0	29_73	0.903
TXNDC2	Thioredoxin domain-containing protein 2	spermatogenesis; cell differentiation	Testis	7.1	29_246	0.898
TPPP2	Tubulin polymerization-promoting protein family member 2	spermatogenesis; cell differentiation	Testis	8.2	29_73	0.888
AKAP3	A-kinase anchor protein 3	blastocyst hatching	Testis	7.3	29_39	0.880
TNP1	Spermatid nuclear transition protein 1	spermatid development; spermatogenesis	Testis	13.1	29_39	0.874
TSSK6	Testis-specific serine/threonine-protein kinase 6	multicellular organism development; sperm chromatin condensation	Testis	8.2	29_246	0.869
MAEL	Protein maelstrom homolog	spermatogenesis	Testis	7.3	29_39	0.864
TCP10L	T-complex protein 10A homolog 1	transcription corepressor activity	Testis	8.1	29_39	0.864
CCIN	Calicin	spermatogenesis	Testis	7.3	29_73	0.862
PRAME	Melanoma antigen preferentially expressed in tumors	cell differentiation	Testis	7.3	30_39	0.860
PRM1	Sperm protamine P1	spermatogenesis; cell differentiation	Testis	14.3	29_73	0.850
PRM2	Protamine-2	spermatogenesis; spermatid development	Testis	14.3	29_73	0.849
GGN	Gametogenetin	spermatogenesis; cell differentiation	Testis	7.5	29_39	0.845
ROPN1L	Ropporin-1-like protein	sperm capacitation	Testis	9.0	29_39	0.844
CABYR	Calcium-binding tyrosine phosphorylation-regulated protein	sperm capacitation	Testis	8.0	29_39	0.840
INSL3	Insulin-like 3	spermatogenesis	Testis	8.9	29_39	0.802
ACRBP	Acrosin-binding protein	spermatid development; acrosome assembly	Testis	9.3	30_246	0.798
PHOSPHO1	Phosphoethanolamine/phosphocholine phosphatase	regulation of bone mineralization	Testis	7.3	29_246	0.782
SPATA24	Spermatogenesis-associated protein 24	spermatogenesis; cell differentiation	Testis	7.5	29_73	0.778
SPINK2	Serine protease inhibitor Kazal-type 2	spermatid development; acrosome assembly	Testis	9.2	29_73	0.773
PCSK4	Proprotein convertase subtilisin/kexin type 4	sperm capacitation	Testis	8.2	30_39	0.773
PRSS21	Testisin	spermatogenesis	Testis	7.0	30_168	0.773
NKX2-1	Homeobox protein Nkx-2.1	thyroid gland development	Thyroid	8.5	29_202	0.870
TG	Thyroglobulin	thyroid gland development	Thyroid	12.3	30_93	0.869
PAX8	Paired box protein Pax-8	thyroid gland development	Thyroid	10.4	29_499	0.802
FOXE1	Forkhead box protein E1	thyroid gland development	Thyroid	7.5	27_376	0.778
TRIP6	Thyroid receptor-interacting protein 6	chordate embryonic development	Uterus	7.6	12_209	0.762
