# Supplementary material for: Identifying transcriptomic correlates of histology using deep learning
Source: PLoS One. 2020 Nov 25;15(11):e0242858. doi: 10.1371/journal.pone.0242858 (PMC7688140; doi:10.1371/journal.pone.0242858)
Supplement: S1 File — (PDF) [file pone.0242858.s008.pdf]

S1 File. Supporting information.

Selection of representative whole slide image tiles

Unfortunately, guided backpropagation visualization cannot be applied exhaustively because it would involve an unmanageably large number of feature-histological image (f,X) pairs. To deal with this problem, we developed an algorithm for selecting a small number of representative histological image tiles to be visualized with guided backpropagation.

select k=3 representative samples for each tissue:

consider the (gene, feature, tissue) table (g,f,t) with corr(g,f) $\geq$ 0.8 and highest median tissue log_2 gene expression $\geq$ 10

for each tissue t

get the samples of the given tissue: samples(t)

get the features f such that (f,t) appear in the table

construct the matrix val(f,s) = the value of the feature f in sample s, for s in samples(t) and f such that (f,t) in table

normalize the rows of this matrix: val(f,s) = val(f,s) / $\text{norm}_{s'}$ val(f,s')

select the samples with the k largest values of $\min_f$ val(f,s) as representative samples for tissue t: selected_samples(t) = k-argmax_s $\min_f$ val(f,s)

Note that $\min_f$ val(f,s) ensures that if the sample s is chosen as representative for tissue t, then all features f associated to this tissue have a value at least $\min_f$ val(f,s).

The same algorithm is applied for selecting a single representative tile from each sample.

Guided backpropagation image normalization

Guided backpropagation generates images of gradients X that must be normalized for visualization. We use the following log_2 transformation of the gradients for better emphasizing the lower intensity details:

$$X_{\log} = \log_2\left(1+\lambda\frac{X^+}{M}\right) - \log_2\left(1+\lambda\frac{X^-}{M}\right)$$

where $X^{\pm} = \frac{|X| \pm X}{2}$, $M = \max\left(\max(X^+), \max(X^-)\right)$, λ=10. In turn, $X_{\log}$ is further normalized to the range [0,1]:

$$X_{norm} = \frac{X_{\log} - \min(X_{\log})}{\max\left(X_{\log} - \min(X_{\log})\right)}.$$
